# Similarity-based search of model organism, disease and drug effect phenotypes

**DOI:** 10.1186/s13326-015-0001-9

**Published:** 2015-02-19

**Authors:** Robert Hoehndorf, Michael Gruenberger, Georgios V Gkoutos, Paul N Schofield

**Affiliations:** Computational Bioscience Research Center, King Abdullah University of Science and Technology, 4700 KAUST, Thuwal, 23955-6900 Saudi Arabia; Computer, Electrical and Mathematical Sciences & Engineering Division, King Abdullah University of Science and Technology, 4700 KAUST, Thuwal, 23955-6900 Saudi Arabia; Department of Computer Science, Aberystwyth University, Llandinam Building, Aberystwyth, SY23 3DB UK; Department of Physiology, Development & Neuroscience, University of Cambridge, Downing Street, Cambridge, CB2 3EG UK

**Keywords:** Phenotype, Semantic similarity, Ontology

## Abstract

**Background:**

Semantic similarity measures over phenotype ontologies have been demonstrated to provide a powerful approach for the analysis of model organism phenotypes, the discovery of animal models of human disease, novel pathways, gene functions, druggable therapeutic targets, and determination of pathogenicity.

**Results:**

We have developed PhenomeNET 2, a system that enables similarity-based searches over a large repository of phenotypes in real-time. It can be used to identify strains of model organisms that are phenotypically similar to human patients, diseases that are phenotypically similar to model organism phenotypes, or drug effect profiles that are similar to the phenotypes observed in a patient or model organism. PhenomeNET 2 is available at http://aber-owl.net/phenomenet.

**Conclusions:**

Phenotype-similarity searches can provide a powerful tool for the discovery and investigation of molecular mechanisms underlying an observed phenotypic manifestation. PhenomeNET 2 facilitates user-defined similarity searches and allows researchers to analyze their data within a large repository of human, mouse and rat phenotypes.

## Background

Our increasing ability to phenotypically characterize genetic variants of model organisms, coupled with systematic and hypothesis-driven mutagenesis efforts, is resulting in a wealth of information about phenotypes. Increasingly, phenotype associated information is represented using ontologies [1], and methods for systematic analysis of phenotypes need to utilize the knowledge contained in these ontologies [2]. One successful analysis approach, leveraging ontologies, is the use of semantic similarity, which applies a similarity measure between terms in phenotype ontologies so as to compute the phenotypic similarity between entities that are represented by them [3]. Phenotypic similarity between different biological entities can be indicative of a large number of biological relations that span multiple scales, and can be effectively utilised so as to reveal gene function [4], mutations underlying genetically-based diseases [5-8] as well as drug-target relationships [9].

One challenge in making these analysis methods and results available to a wide range of researchers is the complexity involved in preparing the underlying data and the time required to perform the analysis. We have developed PhenomeNET 2, a system that provides a web-based interface to perform similarity-based searches over a large repository of phenotypes. PhenomeNET 2 is based on the PhenomeNET platform which pre-computes similarity between a wide range of model organisms, diseases and drug effect profiles, but does not allow searches based on user-specified phenotype profiles. PhenomeNET 2 can now be used to measure semantic similarity between user-specified phenotypic profiles and phenotypes observed in rat, mouse, nematode worm, slime mold and fruitfly strains and variants, human diseases and drug-associated biological effects. The PhenomeNET 2 public webserver is available at http://aber-owl.net/phenomenet.

## Implementation

### Overview

**Figure 1:**
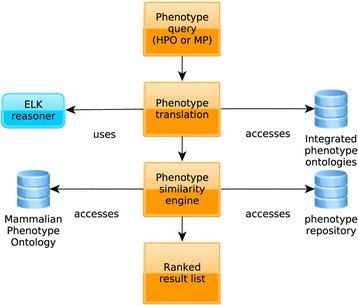
**PhenomeNET 2 analysis and architecture overview.**

Figure 1 provides a high-level overview of the components of PhenomeNET 2. These consist of a frontend, implemented in PHP, and a backend consisting of two parts: an ontology-based phenotype integration service that integrates and translates phenotype ontologies of multiple species, and a similarity service that computes the semantic (phenotypic) similarity between phenotype descriptions.

It was previously only possible to explore the PhenomeNet using genes or their identifiers, or labels or identifiers of diseases that were already included in the network. A key use case for PhenomeNET 2 is the discovery of phenotypically related mutants and diseases using investigators’ own phenotype profiles for searching the network. In order to achieve this, PhenomeNET 2 implements several updates in comparison to the original PhenomeNET system [5]:

- PhenomeNET 2 has a completely novel and updated user interface, which facilitates search of animal model phenotypes, disease phenotypes or drug effect profiles based on combinations of user-specified terms from the MP or HPO;
- PhenomeNET 2 contains a revised phenotype knowledge base over which similarity is computed: additions include phenotypes from the rat model organism database [10] and the slime mold model organism database [11], drug effect profiles [9], and disease phenotypes from Orphanet [6]; yeast and zebrafish phenotypes, which were included in the original PhenomeNET knowledge base, were removed in PhenomeNET 2 as they do not use a pre-composed phenotype ontology for characterizing abnormalities in mutants;
- similarity computation has been reimplemented in C++ to improve query performance and reduce the memory footprint.

### Cross-species integration

PhenomeNET 2 accepts phenotype descriptions that correspond to terms that are available from either the Human Phenotype Ontology (HPO) [12] or the Mammalian Phenotype Ontology (MP) [13]. Using the definitions created for phenotype ontologies [14], we have previously developed a method to integrate phenotype ontologies of multiple species into a single framework that can be used to “translate” phenotypes between different species [5]. For this purpose, we integrate species-specific phenotype ontologies based on the formal definitions that have been created for these ontologies [14]. Cross-species integration is achieved by using the species-independent anatomy ontology Uberon [15] and the Gene Ontology [16] to integrate anatomical entities and biological processes and functions across species, and the species-independent ontology of qualities PATO [17] to characterize the type of abnormal phenotypes observed. These ontologies are combined with anatomy ontologies such as the Mouse Anatomy ontology [18] and the Foundational Model of Anatomy [19] using a knowledge-based approach for combining anatomy and phenotype ontologies [20]. A description logic reasoner can then be used to infer sub- and super-class relations across mouse and human phenotype ontologies.

As a new addition, we have added the Dictyostelium Phenotype Ontology [11] to the set of ontologies in PhenomeNET 2. To integrate this ontology, we have added formal PATO-based entity-quality definitions [17] to 505 classes. The definitions we created are available at http://aber-owl.net/aber-owl/dicty/dicty-xp.obo.

In PhenomeNET 2, the integration and inference method is implemented in Java and relies on the OWL API [21] and the ELK OWL reasoner [22]. The integrated phenotype ontology used by PhenomeNET 2, and the source code for performing the ontology integration and reasoning, is freely available from the project’s website.

### Phenotype knowledge base

PhenomeNET 2 utilizes a knowledge base that consists of animal model phenotypes (slime mold, nematode worm, fruitfly, rat, mouse), disease phenotypes (Orphanet and OMIM), and drug effects (SIDER). In comparison to PhenomeNET, we have added drug effect phenotypes (described previously [9]), slime mold and rat phenotypes. To add rat phenotypes, we downloaded the phenotype annotations of rat genes with the MP from the Rat Genome Database ftp://rgd.mcw.edu/pub/data_release/annotated_rgd_objects_by_ontology/rattus_genes_mp and incorporated them in PhenomeNET 2 similarly to mouse phenotypes. In particular, we conjunctively combine the individual phenotype classes and treat this conjunction as a phenotypic representation of the gene within PhenomeNET 2. Using this method, we incorporated 6,464 MP phenotypes annotations to 1,057 rat strains, 1,545 genes and 1,860 rat QTLs.

Similarly, we obtain slime mold phenotypes annotated with the Dictyostelium Phenotype Ontology from DictyBase (http://dictybase.org/db/cgi-bin/dictyBase/download/download.pl?area=mutant_phenotypes&ID=all-mutants.txt) and represent the slime mold mutants as a conjunction of phenotypes.

### Gene–disease association datasets

We use several curated datasets to evaluate the performance of PhenomeNET 2 for prioritizing candidate genes of disease. We use the curated set of gene–disease associations from the Rat Genome Database available at ftp://rgd.mcw.edu/pub/data_release/annotated_rgd_objects_by_ontology/rattus_genes_rdo, where we filter the gene–disease associations and use only those that have a direct annotation with an OMIM identifier. We further use OMIM’s gene–disease associations, and identify the rat ortholog using the orthologs provided by the Rat Genome Database (ftp://rgd.mcw.edu/pub/data_release/RGD_ORTHOLOGS.txt). Finally, we also use the curated mouse disease models from the Mouse Genome Informatics (MGI) database (ftp://ftp.informatics.jax.org/pub/reports/MGI_Geno_Disease.rpt), excluding conditional mutations and assigning a gene–disease association between gene *G* and disease *D* if the genotype annotated with *D* involves a mutation in *G*.

### Similarity-based search

The similarity computation in PhenomeNET 2 is implemented in C++ to improve performance over Java-based implementations. For similarity computation, we use the groupwise similarity measure SimGIC [23], i.e., the Jaccard index weighted with information content of each class. Specifically, information content *I*(*C*) of an ontology class *C* is based on the probability *P*(*X*=*C*) that a genotype or disease annotation *X* in the phenotype knowledge base is *C*:

(1) $$ I(C) = -\log(P(X=C))  $$

Given two complex phenotypes *P* and *R*, where *P* is characterized by the ontology classes *Cl*(*P*)=*P*_1_,…,*P*_*n*_ and *R* is characterized by the classes *Cl*(*R*)=*R*_1_,…,*R*_*m*_, we define the similarity between *P* and *R* as:

(2) $$ sim(P,R) = \frac{\displaystyle\sum\limits_{x\in Cl(R) \cap Cl(P)}I(x)}{\sum\limits_{y\in Cl(R) \cup Cl(P)}I(y)}  $$

where *Cl*(*X*) is the smallest set containing *X* that is closed against the super-class relation in MP, i.e., $Cl(X) = \{x | x \in X\text {or }\exists y:y \in X \land y\sqsubseteq _{\textit {MP}} x \}$ (where $y \sqsubseteq _{\textit {MP}} x$ means that *y* is a subclass of *x* in MP).

Phenotype similarity is computed using only MP terms due to the higher performance in prioritizing candidate genes for diseases using MP [24]. The repository of phenotype descriptions over which similarity is computed consists of the phenotype descriptions available from the Mouse Genome Informatics (MGI) [25], Rat Genome Database [10], WormBase [26], DictyBase [11], Saccharomyces Genome Database [27], Online Mendelian Inheritance in Man (OMIM) [28], Orphanet [29] and SIDER databases [30].

The PhenomeNET 2 interface is implemented in PHP using the Bootstrap CSS stylesheets, and the PhenomeNET 2 interface employs webservices from the Ontology Lookup Service [31,32] at the European Bioinformatics Institute to display ontology structures of the MP and HPO. Information is processed on the webserver in PHP which forwards the user-based query to the Java backend through a Unix socket connection, and receives the response from the Java backend also through a Unix socket connection.

## Results and discussion

We have developed PhenomeNET 2 which extends the PhenomeNET platform and enables similarity-based searches for user-specified phenotype profiles over a repository of animal model phenotypes, human Mendelian diseases and drug effect profiles. Our implementation of PhenomeNET 2 is available at http://aber-owl.net/phenomenet.

**Figure 2:**
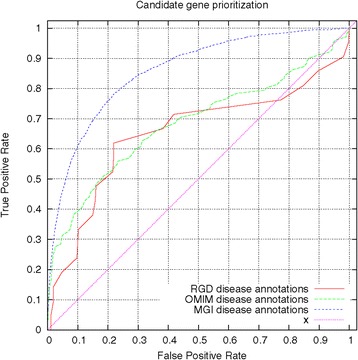
**Performance of candidate gene prediction in PhenomeNET 2.** RGD disease annotations prioritize rat models and use RGD’s disease model annotations as true positives. OMIM disease annotations prioritize rat models and use OMIM’s disease–gene associations as true positives; OMIM genes are mapped to rat genes through orthology. MGI disease annotations prioritize mouse models and use MGI’s disease models as true positives. The ROCAUCs are 0.65, 0.68 and 0.86, respectively.

We evaluated the performance of PhenomeNET 2 for prioritizing candidate genes of disease using rat phenotypes. As rat models are ranked based on their phenotypic similarity to the disease, we use a receiver operating characteristic (ROC) curve [33] to evaluate the results. A ROC curve is a plot of the true positive rate as a function of the false positive rate, and is derived by comparing predicted associations against those asserted in the cognate model organism database. The ROC curve for prioritizing rat disease models as well as mouse disease models is shown in Figure 2. The area under the ROC curve is 0.65 when using gene–disease associations from the Rat Genome Database as evaluation set and 0.68 when using OMIM’s gene–disease associations as evaluation set.

The low recovery of disease annotations from rat models is likely a consequence of the method of annotation used by the Rat Genome Database and the inclusion of very large numbers of olfactory receptor genes in the annotated gene corpus. Of the total 1,545 rat genes annotated to MP, 1,265 are olfactory receptors which each bear a single annotation to *taste/olfaction phenotype* (MP:0005394). Furthermore, the extensive use of electronic inference through orthology, and the separate criteria used for disease and phenotype annotation means that the disease phenotypes and the annotated phenotypes of individual rat models often do not match, i.e., it would be impossible to infer even the domain of the asserted human or mouse diseases from the phenotype annotations for many genes. For example, *Col2a1* (RGD:2375) is annotated only to the *Chondrodystrophy* (MP:0002657) phenotype but to 30 disease classes as varied as *Stickler syndrome*, *Femur head necrosis*, *hypothyroidism* and *myopia* using a disparate range of human disease associations and types of evidence.

To further evaluate query performance and its suitability for real-time user queries, we constructed 1,000 random queries, each consisting of 10 randomly selected MP classes, and performed a similarity-based search across our phenotype repository using the PhenomeNET 2 system. An average query using PhenomeNET 2 system with 10 phenotype terms in the query takes 5.1 seconds to complete. Compared to the Groovy-based implementation of PhenomeNET, this is a 12-time improvement in performance, and this improved performance enables real-time user-specified queries.

There are several further related tools that use similar algorithms and perform similar analyses. In particular, the Phenomizer [34] is a tool for diagnosing patients based on semantic similarity searchers over OMIM diseases using the Human Phenotype Ontology. Phenomizer is implemented in Java and can also perform real-time and user-specified searches. However, it currently uses the Human Phenotype Ontology and is limited to searching diseases available in the OMIM repository, while PhenomeNET 2 uses a larger repository and can search phenotypes across multiple model organism species, diseases and drug effect profiles.

Another related software is PhenoDigm [35], a system similar to PhenomeNET in that it precomputes similarity between model organisms and diseases. PhenoDigm does not currently support user-defined queries over its repository of phenotypes. Finally, functionally the most similar tool to PhenomeNET 2 is the search interface provided by the Monarch Initiative (http://monarchinitiative.org/analyze/phenotypes/). The Monarch Initiative provides the possibility to search mouse and zebrafish models as well as human diseases based on a set of user-specified phenotypes. The main differences to PhenomeNET 2 are the choice of similarity measure and the underlying phenotype knowledge base: the Monarch search tool utilizes the OWLSim tools [7] to compute semantic similarity instead of simGIC used by PhenomeNET 2, uses a single integrated phenotype ontology (the Monarch ontology) instead of a combination of multiple species-specific phenotype ontologies used by PhenomeNET 2, and incorporates zebrafish phenotypes but no fly, worm, slime mold or drug effect phenotypes.

In the future, we plan to incorporate different similarity measures. For example, we intend to experiment with using the Semantic Measures Library (SML) [36] and allow users to select multiple different similarity measures for their search. However, the use of a generic library written in Java will require careful evaluation of query performance.

## Conclusions

Whilst PhenomeNET provides a powerful means to explore the phenomic space occupied by model organisms, human genetic diseases, and pharmacological agents captured in major data resources, PhenomeNET 2 provides the ability to take a newly-derived phenotypic profile from the experimental or genetic manipulation of an organism, or an un-diagnosed patient, and conduct the phenotypic equivalent of a user-defined “BLAST”-type search across a repository of phenotypes. Such a tool is of interest to many communities concerned with phenomics and the analysis of phenotypes. For example, the results of a PhenomeNET 2 search will allow investigators to construct hypotheses about the pathways in which the gene under investigation is involved by looking for closely related phenotypes [37], or, in phenotype-driven studies, prioritize candidate genes in either human or mouse. The ability to search through drug-related phenotypes will also help in the formulation of hypotheses about potential genetic underpinnings of otherwise uncharacterized phenotypes through knowledge of drug targets, or in establishing potential therapeutic strategies where loss of gene function and drug induced phenotypes are concordant.

## Availability and requirements

- **Project name:** PhenomeNET 2
- **Project home page:**http://aber-owl.net/phenomenet and https://code.google.com/p/phenomeblast
- **Operating system(s):** Platform independent
- **Programming language:** Groovy, Java, C++, PHP
- **Other requirements:** Boost library, OWLAPI, ELK reasoner
- **License:** New BSD license
- **Any restrictions to use by non-academics:** none
